# First Is Best

**DOI:** 10.1371/journal.pone.0035088

**Published:** 2012-06-27

**Authors:** Dana R. Carney, Mahzarin R. Banaji

**Affiliations:** 1 Management of Organizations, University of California, Berkeley, California, United States of America; 2 Department of Psychology, Harvard University, Cambridge, Massachusetts, United States of America; Georgia State University, United States of America

## Abstract

We experience the world serially rather than simultaneously. A century of research on human and nonhuman animals has suggested that the first experience in a series of two or more is cognitively privileged. We report three experiments designed to test the effect of first position on *implicit preference* and *choice* using targets that range from individual humans and social groups to consumer goods. Experiment 1 demonstrated an implicit preference to buy goods from the first salesperson encountered and to join teams encountered first, even when the difference in encounter is mere seconds. In Experiment 2 the first of two consumer items presented in quick succession was more likely to be chosen. In Experiment 3 an alternative hypothesis that first position merely accentuates the valence of options was ruled out by demonstrating that first position enhances preference for the first even when it is evaluatively negative in meaning (a criminal). Together, these experiments demonstrate a “first is best” effect and we offer possible interpretations based on evolutionary mechanisms of this “bound” on rational behavior and suggest that automaticity of judgment may be a helpful principle in clarifying previous inconsistencies in the empirical record on the effects of order on preference and choice.

## Introduction

*You walk into a room and first meet Maxine, then Max.*

*In a grocery store, your eyes first notice Bosc pears, then Bartlett pears.*

*Your stockbroker first tells you about a new stock option Bentametrix, then Mentametrix.*

In these examples, as in so much of life, information is experienced sequentially. What is the effect of an item's position on preference for it? There is no reason, at least not a rational one, for preference to be guided by the order in which two items in a series are encountered. Max and Maxine are equally likely to be good people, until we know otherwise; Bartlett pears are no less good than Bosc, but depend on the recipe; and Mentametrix is just as likely to be a smart stock choice as the alternative–in the absence of other information.

It is inherent in the nature of experience, unfolding as it does in time, to encounter events sequentially [1]. Choices and preferences, if they are to maximize subjective expected utilities, ought to be based on rational dimensions of choice such as the quality and value of the object but not on the position in which the option was encountered. However, as the last half-century of research on judgment and decision making has shown, judgments and decisions made under uncertainty are boundedly rational [2]–[6]

### The Power of Primacy

In humans and other animals, we know that primacy has power with undue emphasis placed on the first instance that is encountered. What is experienced first is remembered better [7]–[10], it drives attachment more strongly [11]–[13], creates stronger association with the self [14], influences impressions more decisively [15]–[18], and persuades more effectively [19]–[21].

Literally gravitating toward the first physical object encountered appears to be present in the earliest forms of attachment across species. For example, research on the imprinting process in baby chicks shows that the first object the newborn sees–whether it be another animal or inanimate object–is more likely to become the object of attachment [11]. In adult humans, we know that the first argument presented on even complex topics has greater persuasive appeal and is more likely to change minds [19].

Although first seems to exert influence in such moments, little is known about the choices that adult humans make of everyday people, objects, and events and the role of first position. In fact, in work with adult humans, results are sometimes mixed [19]. We propose that judgments that are relatively devoid of conscious awareness will consistently reveal an effect in which firsts are considered best because firsts are privileged for several–very basic–reasons that heuristic processes may rely on. In particular, it is possible that the evidence which does exist on psychological processes close to attachment and preference may show an even more robust effect if the method by which preferences are elicited can circumvent conscious awareness by reliance upon automatic rather than deliberative cognitive processes. Thus, the goal of the current report was to directly test whether firsts would be consistently preferred on automatic measures of preference and choice–even when firsts may or may not be preferred on deliberative measures of preference and choice.

## Experiment 1

In Experiment 1 we tested the “first is best” hypothesis using three different pairs of stimuli: two male salespersons, two female salespersons, and two teams. Participants were given each of the items of a pair in sequence and then participants indicated their preference.

### Method

#### Participants and procedure

One hundred twenty-three participants were presented with three different choice-pairs and then probed for preference after each pair. Participants were introduced to a pair of teams they may wish to join (the “Hadleys” and the “Rodsons”), a pair of male salespersons from whom they may purchase a car (“Jim” and “Jon”), and a pair of female salespersons from whom they may purchase a car (“Lisa” and “Lori”). Four photos of each individual (e.g., 4 different photos of “Lisa”) and 4 different team member names (e.g., 4 different members of the “Hadley” team) were shown for a total of 24 stimuli. Team-member names were balanced for word-length and letter-usage and faces were balanced within pair for attractiveness and emotional expression. Each option within a pair was presented sequentially for 30-seconds and participants were forced to maximally consider both options. Immediately after each choice-pair was presented, participants completed a measure which assessed automatic preference for each option (an Implicit Association Test, or IAT) [22]. Self-reported preference was also measured. Order of choice-pairs was randomized as was which option was presented first. All of this research (Experiments 1–3) was approved by the human subjects review board at Harvard University. Written and signed informed consent was obtained from participants in Experiments 1 and 3 and verbal consent was obtained from the field participants in Experiment 2.

#### Preference measures

The measure of automatic preference was an evaluative IAT for each choice-pair. In these tests, each category label–in this case, team names and salespersons–were paired with “Better” and “Worse.” For example, the IAT assessed the degree to which Lisa versus Lori (and also Jim vs. Jon and Hadleys vs. Rodsons) were spontaneously associated with positive and negative attributes, with order of first paired block (Lisa + Better; Lori + Worse) counterbalanced. The difference in average response latency to the paired blocks was divided by the pooled SD yielding an index of implicit preference for one person (or team) versus the other (the score is known as a D-score) [22]. In the IATs, the items for the salespersons were 4 photographs of each individual or team. The items for “Better” and “Worse” were roughly synonyms for the words (e.g., wonderful, best; horrible, worst).

Deliberative, self-reported preference was measured with: (a) a 7-point scale labeled “I strongly prefer Lisa to Lori,” to “I strongly prefer Lori to Lisa”; (b) two “feeling thermometers” of Lisa and Lori preference from 0 (*cold*) –100 (*warm*); the difference between the two thermometers was z-scored and averaged with the z-scored 7-point rating item (all option-pairs were rated on these self-reported items). Both self-report and implicit measures contained a midpoint of zero indicating no preference. Order of preference-measures (implicit vs. explicit) was counterbalanced.

### Results and Discussion

**Figure 1:**
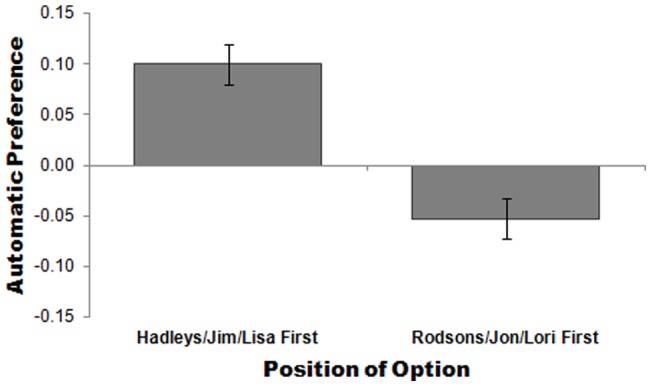
The effect of presentation order on automatic preference for Hadleys/Rodsons, Jim/Jon, and Lisa/Lori. Higher scores indicate a preference for Hadleys, Jim, and Lisa (vs. Rodsons, Jon, and Lori); lower scores indicate a preference for Rodsons, Jon, and Lori (vs. Hadleys, Jim, and Lisa). Error bars are standard errors of the mean.

Repeated measures ANOVA across choice-pairs revealed a main effect of primacy on automatic preference. Automatic preference for first items presented was compared with automatic preference for second items presented (across choice-pairs: Hadleys & Rodsons, Jim & Jon, and Lisa & Lori). Regardless of the actual option, the one presented first compared to the one presented next was significantly more strongly associated with the concept “better” rather than “worse”, *F*(1, 121)  = 20.20, *p*<.001; effect size *r* = .38 (Figure 1). There was no difference in self-reported preference for firsts versus seconds, *F*(1, 121)  = .08, *p* = .78.

## Experiment 2

The IAT is a very particular kind of test of automatic preference, and in Experiment 2 we introduced a different and more realistic measure of preference in the form of a direct choice of a consumer good. When choosing between two pieces of similarly packaged and flavored bubble gum, we either (a) imposed upon participants a time constraint that forced a spontaneous and immediate decision, or (b) allowed a deliberative choice with more time available. Consumer items varied in no way other than serial position; choice of gum was the dependent variable.

### Method

#### Participants and procedure

Two-hundred seven participants were recruited from a train station in Boston, MA. Adults sitting alone were approached by an experimenter who was blind to the hypothesis. Participants were asked to participate in a study on consumer choice (<1% declined). For remuneration they were offered the small consumer item they chose. Two pieces of similar-looking bubblegum (1 piece of “Bubble Yum” and 1 piece of “Bubblicious;” equal in size and shape) were placed sequentially on a white clipboard. First placement of gum brand and side of clipboard (left vs. right) were counterbalanced. After rapid sequential placement of the two items, participants reached out and grabbed (and kept) their choice.

In the rapid decision task participants were instructed to make their selection fast “within one second or so” whereas in the deliberative decision task they were instructed to select “after you have thought about it.”

### Results and Discussion

**Figure 2:**
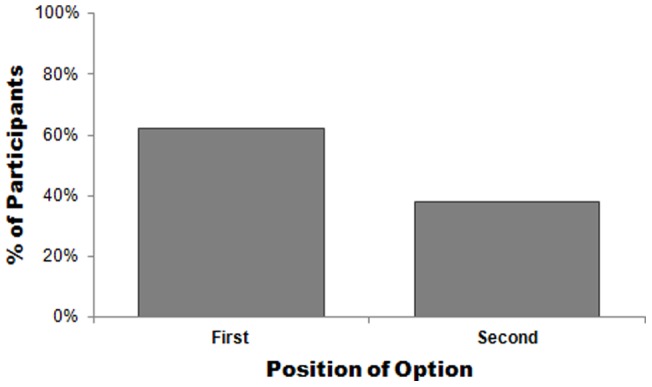
The effect of presentation order on actual choice for bubblegum presented first versus second.

A 2×2 *χ* ^2^ tested the effect of order (first vs. second) on choice for each deliberation condition (automatic vs. controlled) separately. Consistent with results from Experiment 1, participants in the rapid decision task chose the chewing gum presented first (62%) significantly more often than the gum presented second (38%) whereas, participants in the deliberative choice condition were equally as likely to choose the gum presented first (51%) or second (49%): *Χ* ^2^(*df* = 1, *N* = 113)  = 6.53, *p*<.02; effect size *r* = .24 (Figure 2).

Experiments 1 and 2 suggest that firsts are preferred when the choice bypasses deliberative thought across several target domains (individuals, groups, consumer goods) using two measures of preference (the IAT and simple choice). In all cases, preferences were obtained in the automatic choice condition and not in the more consciously controllable response. However, both Experiments 1 and 2 leave open a possibility that challenges the “first is best” hypothesis: It is possible that the first item in a pair does not engender greater preference but a more extreme evaluative response to whatever the existing valence of that object. In Experiments 1 and 2, the stimuli were mildly or extremely positive objects and the observed effect may simply reflect an enhancement of existing valence. In other words, had the first item been obviously aversive, the result could be a stronger negative reaction to the first, resulting in a choice of the second. Experiment 3 was conducted to test this alternative explanation of polarization.

## Experiment 3

We measured automatic preference for negatively valenced options: two criminals convicted of violent crimes. If “first is best,” the first criminal presented should be seen as *more* worthy of parole; if polarization is the effect, the first criminal presented should be viewed as *less* worthy of parole.

### Method

#### Participants and procedure

Two criminals' photographs, from the Florida Department of Corrections website (www.dc.state.fl.us), were used. Photos depicted 29 year-old males known to have committed the same violent crimes. Criminals were wearing identical correctional facility outfits; photos were pre-tested to be equally attractive and both expressing neutral facial expressions. The two photos were selected from a larger pool of 17 photos taken from the same website. Two coders (D. R. C. and B. M.) coded all photos for (1) facial expression from −3 (*extremely negative*) to +3 (*extremely positive*), and (2) attractiveness from −3 (*extremely unattractive*) to +3 (*extremely attractive*). The two photos for which the two coders were in 100% agreement were selected for use.

Thirty-one participants learned that evidence suggests people can make accurate judgments of others after limited exposure to them and were asked to evaluate two criminals and to determine who should “stay in jail” versus “be released on parole.” Participants were shown two criminals, one after the other (order of photos was counterbalanced across participants). Immediately upon seeing the faces of the criminals (named Jim and Jon), a measure of automatic preference assessed participants' speed of associating Jim and Jon with “Worse for Parole” and “Better for Parole” using the same procedure as in Experiment 1. Self-reported preference was also measured.

### Results and Discussion

**Figure 3:**
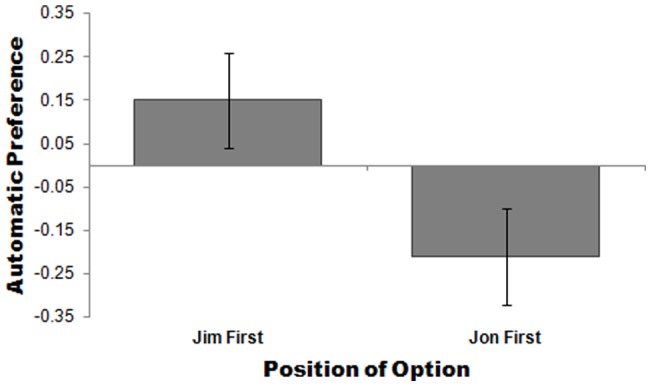
The effect of presentation order on automatic preference for Jim the criminal versus Jon the criminal. Higher scores indicate a preference for Jim (vs. Jon); lower scores indicate a preference for Jon (vs. Jim). Error bars are standard errors of the mean.

Even when the stimuli were evaluatively negative (*imprisoned criminals convicted of violent crimes*) and the judgment called for was evaluatively negative (*who should remain in prison?*), participants automatically associated the first criminal with being more worthy of parole (rather than prison) compared to the second criminal. Regardless of which photo was presented first, it was the one presented first who was judged to be more worthy of parole, *F*(1, 29)  = 4.31, *p*<.05; effect size *r* = .36 (Figure 3). Replicating Experiments 1 and 2, no effects were observed on self-reported preference. On the self-reported measure of preference the means were consistent with those of the implicit results: When Jim was seen first he was preferred for parole over Jon (*M* = .25) and when Jon was shown first he was preferred over Jim (*M* = −.21). However, this effect was not statistically significant: *F*(1, 29)  = 1.88, *p*>.18.

## General Discussion

In three experiments, with tests using options from five different social and consumer-item categories (female salespeople, male salespeople, teams, pieces of gum, and criminals), we obtained a consistent result that on a deep, automatic level of human cognition, firsts are consistently preferred and chosen. Despite the research demonstrating how firsts influence many aspects of human and animal cognition, the six published reports on adult humans testing the effect of order on preference have reported somewhat mixed results [4], [17], [23]–[26].

Mantonakis et al. attempted to explain the mixed results by advancing the idea that expertise moderates the effect of primacy on preference. They argued that expert knowledge and deliberation about options trump locally available heuristics such as order. While their account does explain their own and some others' findings, it does not explain why Bruine de Bruin's work [23], [24], Li and Epley [25], and Nisbett and Ross [4] sometimes found no effect of order and/or other times recency effects. The expertise account alone also doesn't explain the results from the three experiments presented here.

We tested whether firsts would show direct effects on preference and choice using measures of automatic and deliberative cognition and in all three experiments our results suggest it is on measures of automatic cognition that firsts may deliver their impact more consistently. In retrospect, that is probably why existing research, mainly with non-humans, showed an early and automatic tendency to prefer firsts; later work with adult humans which sometimes produced mixed results may have simply been a result of mixed methods, both automatic and controlled, being utilized. Although such a conclusion from the review of existing research may have been possible, it is experiments that use both forms of measuring preferences that can be most convincing. Consistent with our results and the theoretical argument advanced by Mantonakis et al. [26], we propose that judgments that are relatively devoid of conscious awareness will consistently reveal an effect in which firsts are considered best because firsts are privileged for several reasons that heuristic processes may rely on. We note here that accounts of satisficing are consistent with our notion of an automatic preference for firsts–however we refer to automatic preferences resting below the reaches of introspective access and fully developed only after maximizing – in other words, only after both options have been fully presented and considered.

It may well be that a preference for firsts has its origins in an evolutionary adaptation favoring firsts. Research on filial imprinting in non-human animals suggests that attachment to the first may have evolved as an adaptive mechanism to help organisms rapidly discriminate between those entities that are safe versus dangerous [11]. Research on humans' innate preparedness to prefer their own (mother, family, social group), which is also the one encountered first, may account for the potential adaptive utility of a preference for primacy. A primacy effect on preference may have derived from positivity attributed to first experiences not leading to harm. Automatic measures of preference are able to tap into these more primitive human systems which is why the primacy effect can be consistently harvested using such measures.

It may also be that a primacy effect on preference evolved from observations of phenomena like “pecking order” (we thank Joshua Greene for this suggestion). The animal that has alpha status gets to eat first, the person of highest privilege in a family or group is served first (kings, fathers, guests). Perhaps a preference for the first conferred an advantage leading a preference for first to have been a quality favored in natural selection.

In contrast, when controlled processing is possible, other influences can (as they rationally should) override the automatic reliance on the first. This is both the discovery of the present experiments and it also may explain the sometimes mixed outcomes of previous research.

As a pointer to the mechanism by which firsts may have their power, we note that research on comparative judgments shows that whatever is set as the “gold standard” against which others are compared is itself strongly preferred [27], [28]. Although order effects have not been shown to account for direction of comparison findings [27], the basic thrust of this and related work on similarity judgments [29], [30] suggests that firsts may automatically be viewed as the gold standard against which others are to be compared.

Future research will involve further tests of the generality of this phenomenon in different samples and species because such tests are crucial to understanding the nature of this surprising and seemingly important effect. If some of the above speculations are correct, this effect should emerge in infants and young children, as well as in other primates and animals–regardless of whether deliberation was “allowed” or not. Likewise, tests are needed to unlink this primacy effect from other primacy producing results. For example, disambiguating this result from primacy effects in memory can be conducted by having later items in a series repeated more often (to enhance recency) and test whether even under such conditions a preference for first is retained (we thank Jonathan Schooler for this suggestion). Two of our tests (Experiments 1 and 3) were optimized to yield recency effects because when forced to maximally consider all options people tend to choose the most recent item; thus, Experiments 1 and 3 served as extremely conservative tests of our hypothesis (we thank an anonymous reviewer for pointing this out). However, additional tests utilizing paradigms more traditionally optimized to reveal recency (e.g., time delays between first and second options as is the case in persuasion research) should examine whether recency effects are an emergent property of only deliberative cognition–perhaps an indirect measure (such as those used in the three experiments presented here) would demonstrate primacy while self-report measures continue to reveal recency. In the meantime, decision theorists would do well to build in tests of “first is best” in research that will teach us about the impact, boundary conditions and mechanisms of the automatic preference for firsts observed here.
